# Supplementary material for: FUBP1 and FUBP2 enforce distinct epigenetic setpoints for MYC expression in primary single murine cells
Source: Commun Biol. 2020 Oct 1;3:545. doi: 10.1038/s42003-020-01264-x (PMC7530719; doi:10.1038/s42003-020-01264-x)
Supplement: Supplementary file 1 — Supplementary Information [file 42003_2020_1264_MOESM1_ESM.pdf]

a

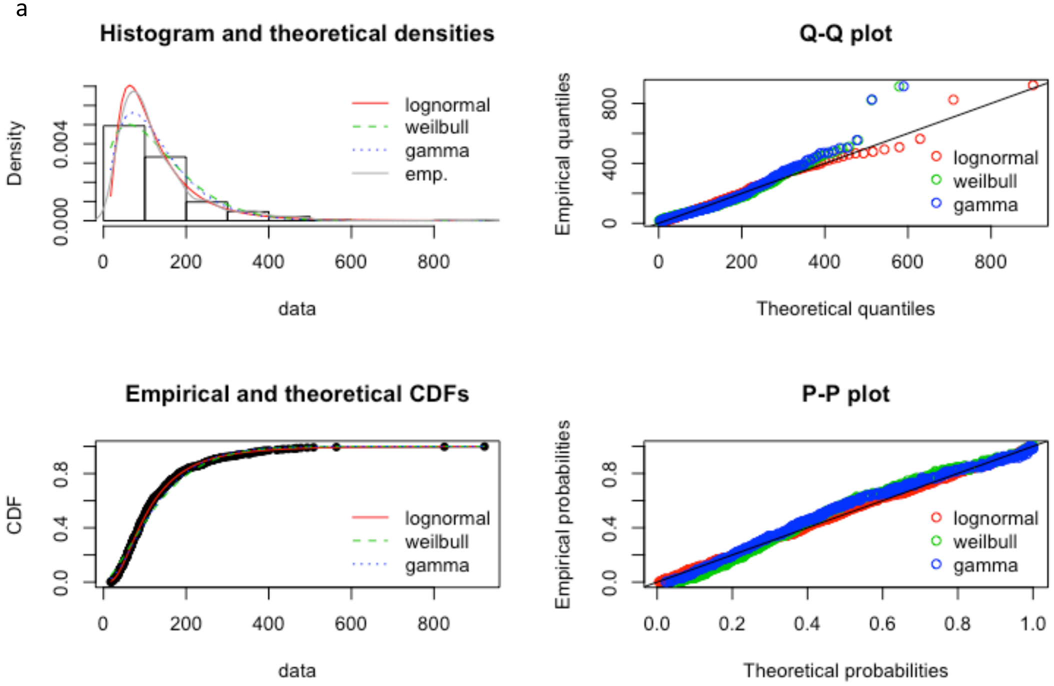

b

```
Goodness-of-fit statistics
                                  lnorm    weibull      gamma
Kolmogorov-Smirnov statistic 0.03037057 0.08827246 0.08082785
Cramer-von Mises statistic   0.05573098 1.19553589 0.80349630
Anderson-Darling statistic   0.37001956 7.73195668 4.60637049

Goodness-of-fit criteria
                                  lnorm  weibull    gamma
Akaike's Information Criterion 5839.048 5933.885 5890.646
Bayesian Information Criterion 5847.525 5942.362 5899.122
```

Supplementary Figure 1. MYC Expression Level in Single MEF May Fit Lognormal Distribution. Distribution of Myc mRNA expression in single MEF were fitted to lognormal, Weibull and gamma distribution. (a) The empirical and theoretical PDF plot (upper left), CDF (lower left), Q-Q plot (upper right), and P-P plot (lower right) are shown. (b) The Goodness-of-fit statistics of the three fittings.

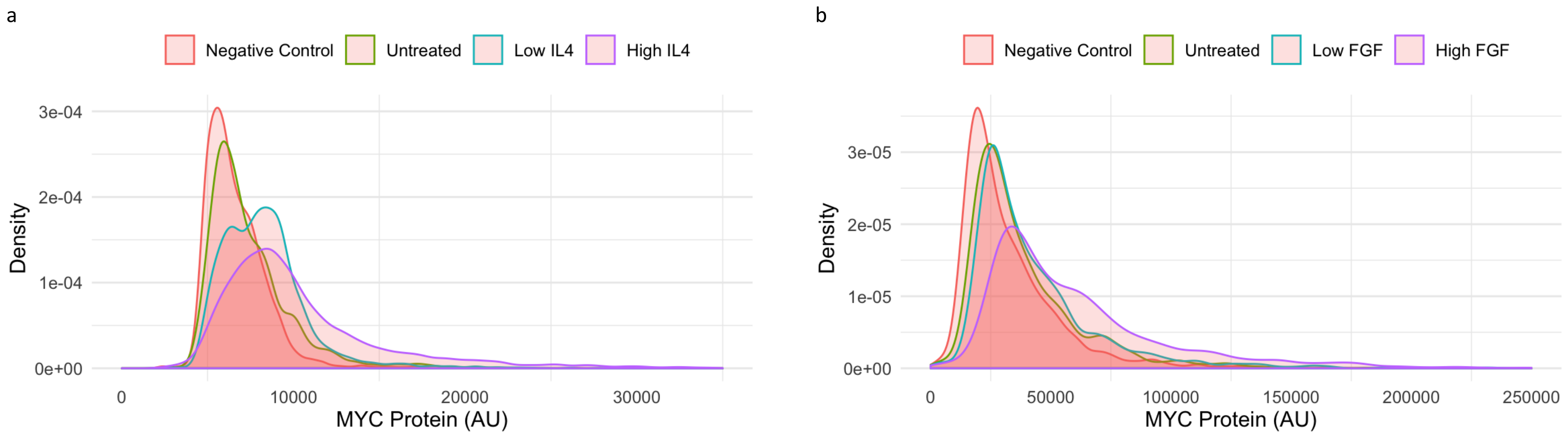

Supplementary Figure 2. Density Plot of MYC Total Nuclear Protein Level in Naïve and IL-4-stimulated B Cells (a), and in Steady-state and FGF- stimulated MEFs (b) are Shown. Low IL-4: 5 ng/ml; high IL-4: 50 ng/ml. Low FGF: 1 ng/ml; high FGF: 4 ng/ml. AU: arbitrary unit. Negative control: non-specific IgG isotype control.

a

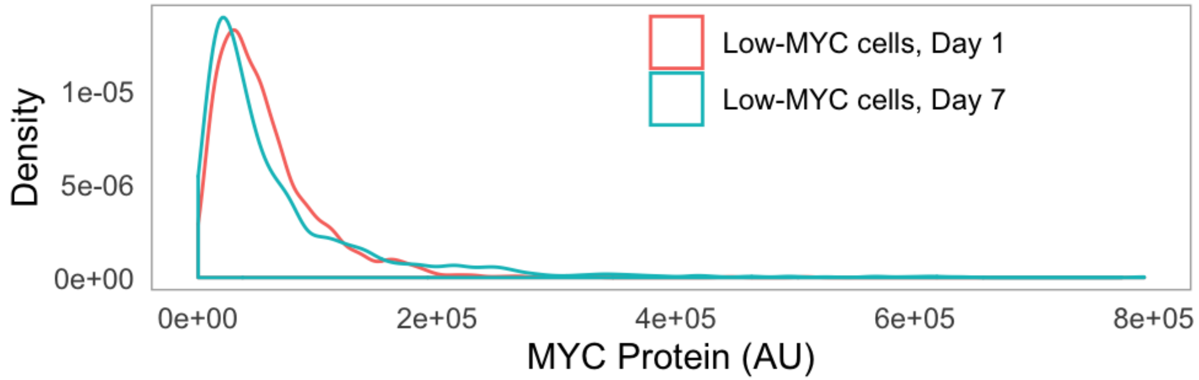

b

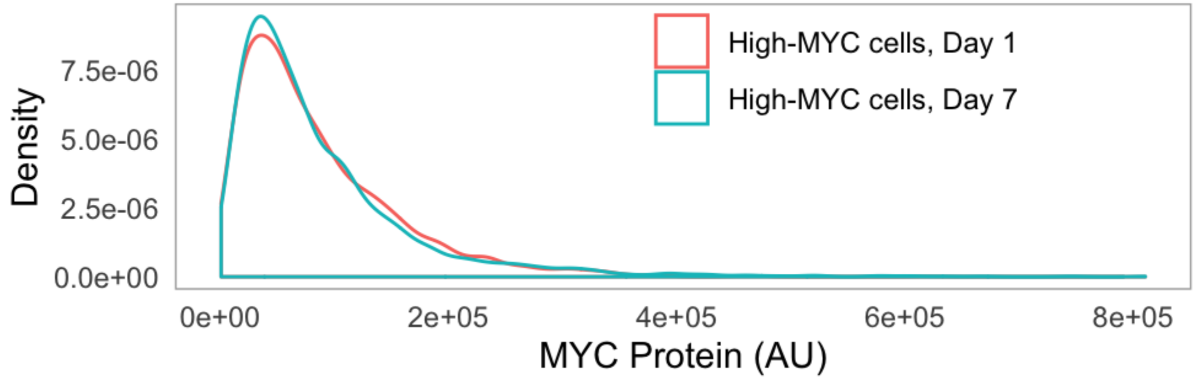

Supplementary Figure 3. The Distribution of MYC Levels in Low-MYC Cells Increased Slightly, while the Distribution of MYC Levels Remained Unchanged in High-MYC Cells after 1 Week of Culture. The density plot of MYC levels in the low- (a) or high-MYC (b) cells on day 1 or 7 after sorting were shown. AU: arbitrary unit.

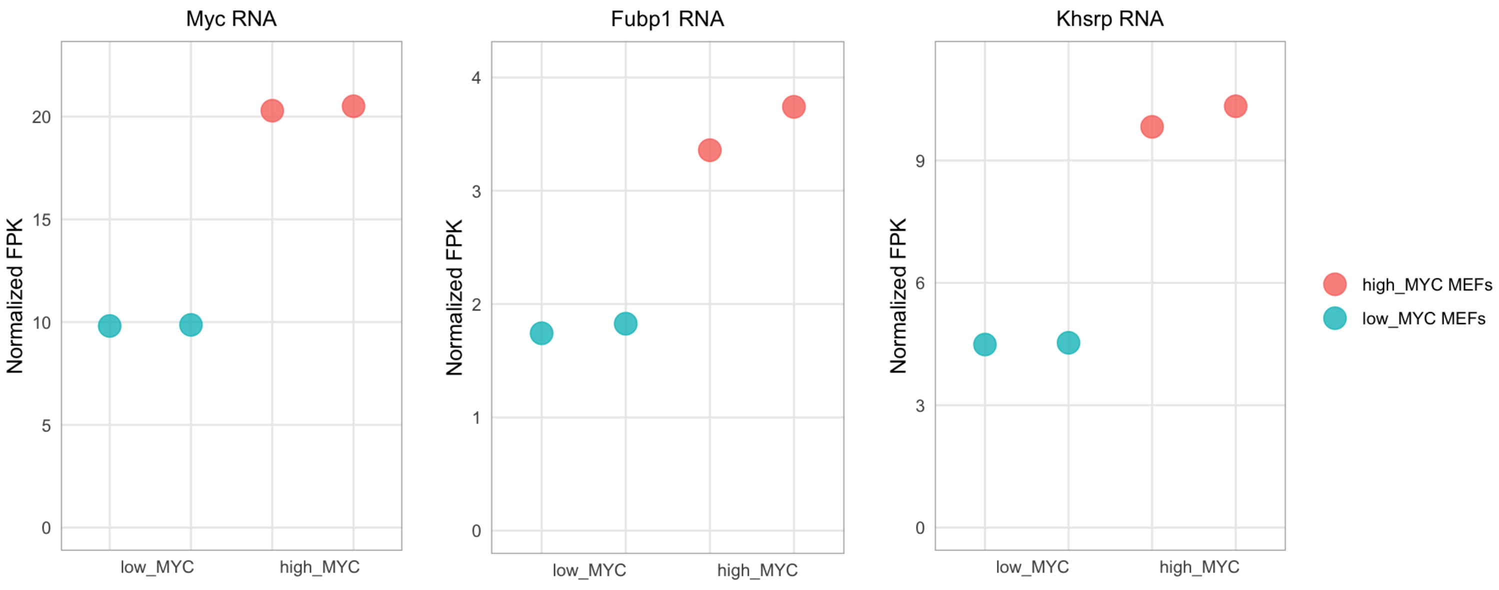

Supplementary Figure 4: *Myc*, *Fubp1* and *Fubp2/Khsrp* Levels at Low- and High-MYC MEFs. The Myc, Fubp1 and Fubp2/khsrp mRNA levels from RNA-seq of low-MYC and high-MYC cells were shown. Each of the low-MYC and high-MYC RNA-seq samples has two technical replicates.

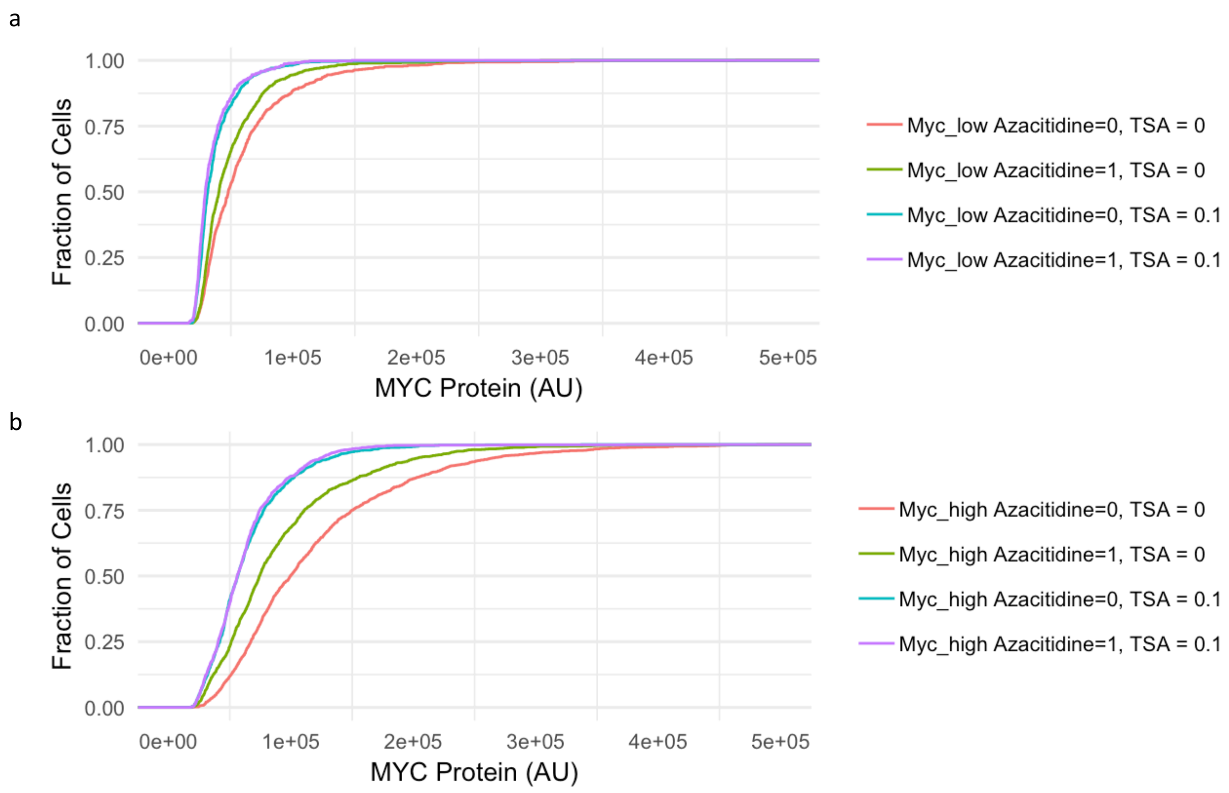

Supplementary Figure 5. Treatment with DNA Methylation Inhibitor 5-azacytidine and/or HDAC Inhibitor Tricostatin A (TSA) Decreases MYC Expression in Both Low- and High-MYC Cells. The low- (a) or high-MYC (b) cells were treated with 5-azacytidine or TSA for 24 h, and the empirical CDF plot of MYC levels were shown. Unit of inhibitor concentration: µM. AU: arbitrary unit.

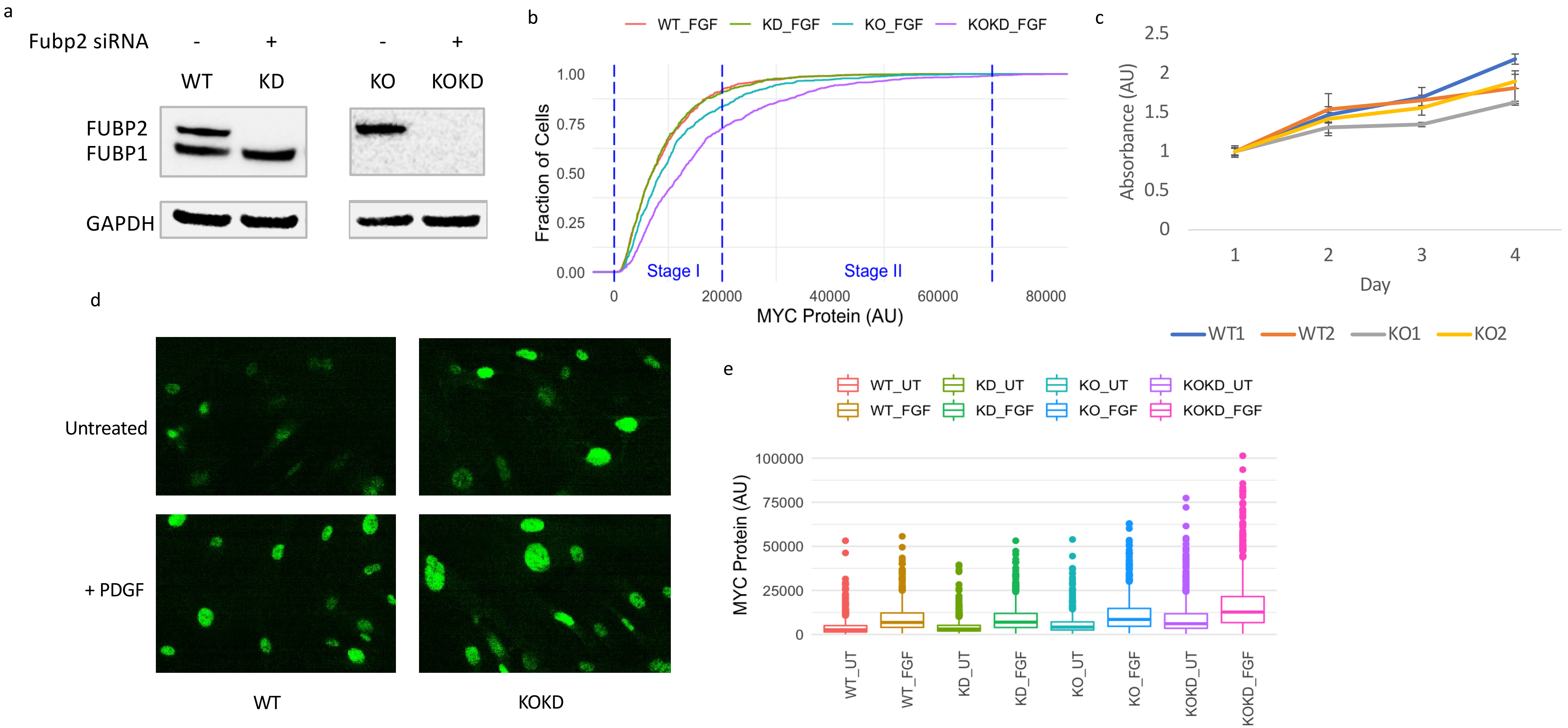

Supplementary Figure 6. Loss of FUBP1 and FUBP2 Causes Increased MYC Levels and Variations in Steady-State (Untreated) and Stimulated Cells. (a) Validation of *Fubp2* Knockdown. The Fubp1 wildtype and knockout MEFs were transfected with non-target siRNA or Fubp2 siRNA for 48 h and followed by western blot analysis. (b) The empirical CDF plot of MYC protein level in FGF-stimulated WT, KD, KO and KOKD MEFs are shown. (c) Proliferation of WT and KO MEFs Determined by WST-1 Assay. Two biological replicate of WT or KO MEFs were examined, and experiments were performed in triplicate. AU: arbitrary unit. (d) The WT and KOKD cells were untreated or treated with PDGF, and representative images of MYC immunostaining were shown. AU: arbitrary unit. (e) The box plot shows loss of FUBP1 and FUBP2 causes increased MYC protein levels and variations in untreated and FGF-stimulated cells. Two ng/ml of FGF were used for treatment. Experiments were repeated with similar results.

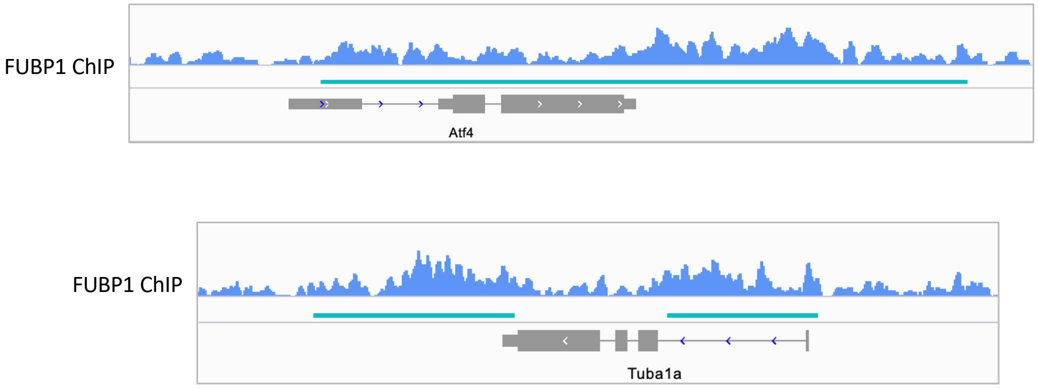

Supplementary Figure 7. FUBP1 Binds Broadly Across *Atf4* and *Tuba1a* Genes. These are examples of genes with a FUBP1 binding pattern similar to FUBP1 binding to *Myc*. Peaks called by SICER algorithm against input control are marked by green line. FDR < 0.01.

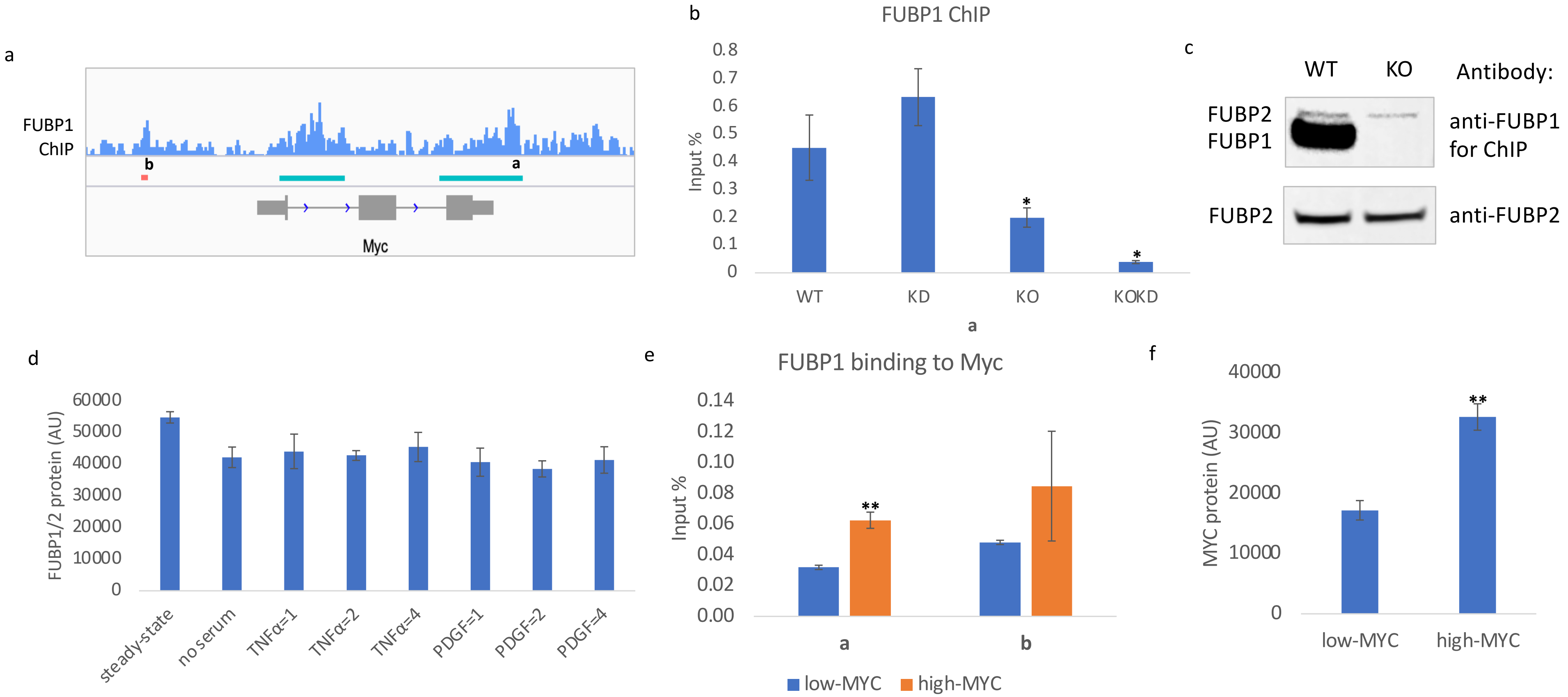

Supplementary Figure 8. The Binding of FUBP1 to the *Myc* Locus is Higher in High-MYC Cells Than in Low-MYC Cells. (a) The FUBP1 ChIP-seq at *Myc* locus in MEFs. The positions of the primers used in Figures b and e were marked with the corresponding letters in this figure. (b) FUBP1 ChIP-qPCR on the *Myc* Locus of WT, KD, KO and KOKD Cells. The positions of the primers used in this figure are marked with the corresponding letters. *, p<0.01, T-test assuming unequal variance, compared with the wildtype sample. (c) Some cross-reaction of the anti-FUBP1 antibody for ChIP (Abcam #ab181111) with FUBP2. The cell lysates of Fubp1 wildtype and knockout MEFs were immunoblotted with anti-FUBP1 antibody for ChIP and anti-FUBP2 antibody. (d) The quantitative immunostaining results of FUBP1/2 at steady-state or upon stimulation with PDGF or TNFα stimulation. AU: arbitrary unit. Experiments were performed in triplicate. (e) FUBP1 ChIP-qPCR on the Myc Locus of low-MYC and high-MYC MEFs. The positions of the primers used in this figure are marked with the corresponding letters in Figure a. (f) The mean MYC protein levels in the low- or high-MYC MEFs are shown. Experiments were performed in triplicate. AU: arbitrary unit. (e-f) **, p< 0.01, T-test assuming unequal variance, compared with the low-MYC sample.

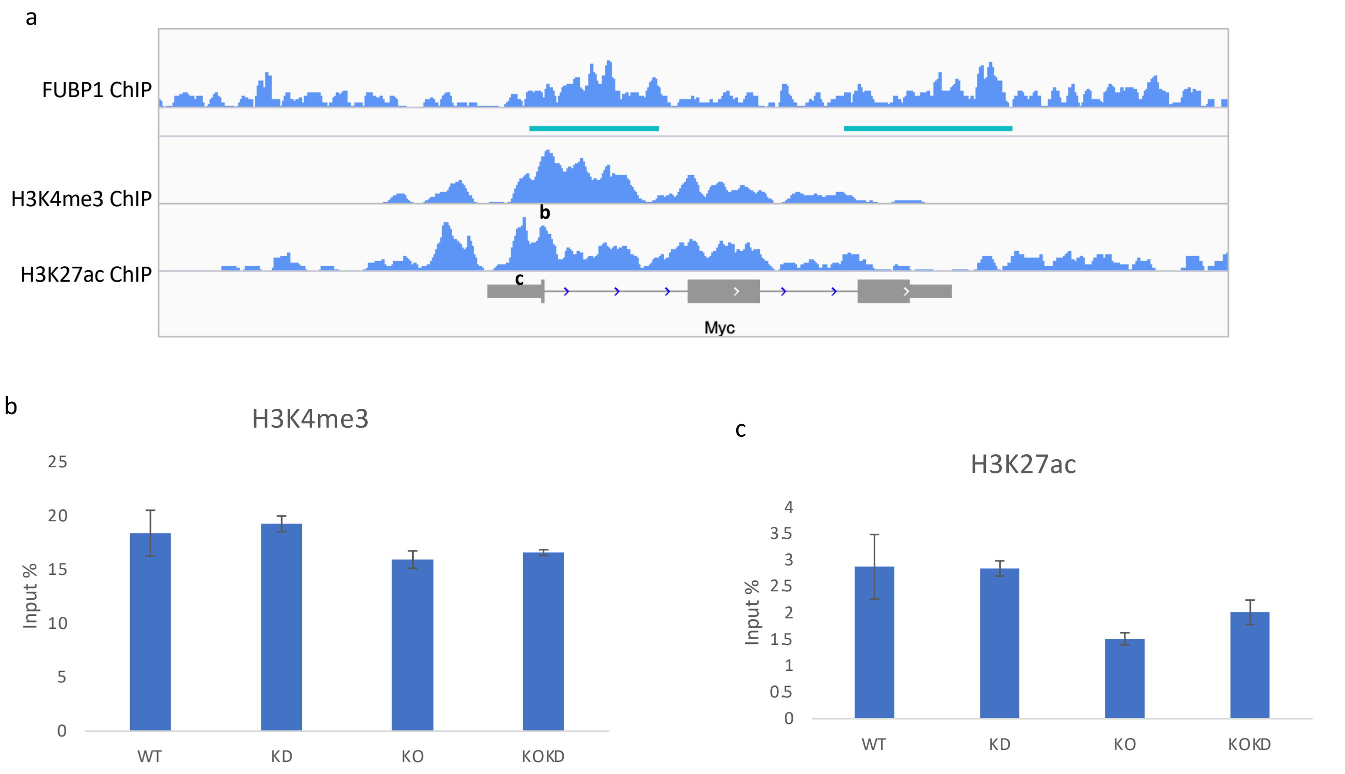

Supplementary Figure 9. H3K4me3 and H3K27ac at the *Myc* Locus in WT, KD, KO and KOKD Cells. (a) First panel: the FUBP1 ChIP-seq at *Myc* locus in MEFs. Second and third panel from top: The ENCODE data of H3K4me3 (ENCFF695BMP) and H3K27ac (ENCFF512EGA) ChIP-seq in MEFs. The positions of the primers used in Figures b and c were marked with the corresponding letters. (b-c) The results of H3K4me3 (b) and H3K27ac (c) ChIP-qPCR on the Myc gene in WT, KD, KO and KOKD MEFs are shown. The positions of the primers used in each figure are marked with the corresponding letters in Figure a. Experiments were performed in triplicate.

ChIP-qPCR	Forward Primer	Reverse Primer	Position in the *Myc* gene
**FUBP1**	GTGAGTTTCAGGCTAGCAAAG	AGGAGTCAGAATGCAAGGCA	Figure 6a, marked by “a”
**FUBP1**	GCGGCACTAGGACTTGATGT	GAACCGCTCAGATCACGACT	Figure 6a, marked by ”b”
**FUBP1**	TCTGCCCTGCCAAAGAACTG	GGACTATCACCTGGGAGCAAG	Figure 6a, marked by “c”
**H3K4me1**	CCGCCTACATCCTGTCCATT	CGAGAGATTCCAGCTCCTCC	Figure 6a, marked by “d”
**H3K4me3**	GAGCTCCTCGAGCTGTTTGA	CTAACCGGCCGCTACATTCA	Supplementary Figure 9a, marked by “b”
**H3K27ac**	GAAGGCAGCTCTGGAGTGAG	GTCCTGGCTCGCAGATTGTA	Supplementary Figure 9a, marked by “c”

Supplementary Table 1. The ChIP-qPCR Primers.
