# Supplementary material for: FUBP1 and FUBP2 enforce distinct epigenetic setpoints for MYC expression in primary single murine cells
Source: Commun Biol. 2020 Oct 1;3:545. doi: 10.1038/s42003-020-01264-x (PMC7530719; doi:10.1038/s42003-020-01264-x)
Supplement: Supplementary file 2 — Description of Additional Supplementary Files [file 42003_2020_1264_MOESM2_ESM.pdf]

Description of Additional Supplementary Files

File Name: Supplementary Data 1
Description: In High-MYC MEFs, Genes with the Highest Fold-Increase (FC>=5) Compared to Low-MYC MEFs (FDR < 0.01) were Enriched for the Inflammatory Response and the Immune Response Genes.

File Name: Supplementary Data 2
Description: Source data for all main figures.
