# Supplementary material for: FUBP1 and FUBP2 enforce distinct epigenetic setpoints for MYC expression in primary single murine cells
Source: Commun Biol. 2020 Oct 1;3:545. doi: 10.1038/s42003-020-01264-x (PMC7530719; doi:10.1038/s42003-020-01264-x)
Supplement: Supplementary file 5 — Reporting Summary [file 42003_2020_1264_MOESM5_ESM.pdf]

nature research

Corresponding author(s): David Levens

Last updated by author(s): Aug 11, 2020

Reporting Summary

Nature Research wishes to improve the reproducibility of the work that we publish. This form provides structure for consistency and transparency in reporting. For further information on Nature Research policies, see Authors & Referees and the Editorial Policy Checklist.

Statistics

For all statistical analyses, confirm that the following items are present in the figure legend, table legend, main text, or Methods section.

n/a	Confirmed	
☒	☐	The exact sample size (*n*) for each experimental group/condition, given as a discrete number and unit of measurement
☒	☐	A statement on whether measurements were taken from distinct samples or whether the same sample was measured repeatedly
☐	☒	The statistical test(s) used AND whether they are one- or two-sided<br>*Only common tests should be described solely by name; describe more complex techniques in the Methods section.*
☒	☐	A description of all covariates tested
☒	☐	A description of any assumptions or corrections, such as tests of normality and adjustment for multiple comparisons
☒	☐	A full description of the statistical parameters including central tendency (e.g. means) or other basic estimates (e.g. regression coefficient) AND variation (e.g. standard deviation) or associated estimates of uncertainty (e.g. confidence intervals)
☒	☐	For null hypothesis testing, the test statistic (e.g. *F*, *t*, *r*) with confidence intervals, effect sizes, degrees of freedom and *P* value noted<br>*Give P values as exact values whenever suitable.*
☒	☐	For Bayesian analysis, information on the choice of priors and Markov chain Monte Carlo settings
☒	☐	For hierarchical and complex designs, identification of the appropriate level for tests and full reporting of outcomes
☒	☐	Estimates of effect sizes (e.g. Cohen's *d*, Pearson's *r*), indicating how they were calculated

Our web collection on statistics for biologists contains articles on many of the points above.

Software and code

Policy information about availability of computer code

Data collection	Image acquisition, automated segmentation, and intensity measurements were performed using Nikon Elements software version 4.30.02.
Data analysis	Data were analyzed and plotted using custom R software.

For manuscripts utilizing custom algorithms or software that are central to the research but not yet described in published literature, software must be made available to editors/reviewers. We strongly encourage code deposition in a community repository (e.g. GitHub). See the Nature Research guidelines for submitting code & software for further information.

Data

Policy information about availability of data

All manuscripts must include a data availability statement. This statement should provide the following information, where applicable:

- Accession codes, unique identifiers, or web links for publicly available datasets
- A list of figures that have associated raw data
- A description of any restrictions on data availability

The RNA-seq, ChIP-seq and TACh-seq sequencing data that support the findings of this study are available in GEO, accession number GSE143800. All other data that support the findings of this study are available from the corresponding author upon reasonable request.

Field-specific reporting

Please select the one below that is the best fit for your research. If you are not sure, read the appropriate sections before making your selection.

☒ Life sciences ☐ Behavioural & social sciences ☐ Ecological, evolutionary & environmental sciences

For a reference copy of the document with all sections, see nature.com/documents/nr-reporting-summary-flat.pdf

Life sciences study design

All studies must disclose on these points even when the disclosure is negative.

Sample size	N/A
Data exclusions	N/A
Replication	Experiments were performed in triplicate.
Randomization	N/A
Blinding	N/A

Reporting for specific materials, systems and methods

We require information from authors about some types of materials, experimental systems and methods used in many studies. Here, indicate whether each material, system or method listed is relevant to your study. If you are not sure if a list item applies to your research, read the appropriate section before selecting a response.

Materials & experimental systems

n/a	Involved in the study	
☐	☒	Antibodies
☐	☒	Eukaryotic cell lines
☒	☐	Palaeontology
☒	☐	Animals and other organisms
☒	☐	Human research participants
☒	☐	Clinical data

Methods

n/a	Involved in the study	
☐	☒	ChIP-seq
☐	☒	Flow cytometry
☒	☐	MRI-based neuroimaging

Antibodies

Antibodies used	Antibodies to MYC (#ab32072), FUBP1 (#ab184111) were from Abcam. Rabbit monoclonal anti-FUBP1 antibody (Abcam, ab181111), anti-H3K4me1 (Abcam, #ab8895), anti-H3K4me3 (MilliporeSigma, #04-745) and anti-H3K27ac (ActiveMotif #39034) were used for the immunoprecipitation.
Validation	The manufacturer's website states they react with mouse proteins and are suitable for the applications we applied. We also validated with western blotting.

Eukaryotic cell lines

Policy information about cell lines

Cell line source(s)	Primary mouse embryonic fibroblasts from gestation day 13.5 were prepared in our lab.
Authentication	N/A
Mycoplasma contamination	N/A
Commonly misidentified lines (See ICLAC register)	N/A

ChIP-seq

Data deposition

☒ Confirm that both raw and final processed data have been deposited in a public database such as GEO.

☒ Confirm that you have deposited or provided access to graph files (e.g. BED files) for the called peaks.

Data access links<br>*May remain private before publication.*	GEO GSE143800
Files in database submission	See GEO
Genome browser session<br>(e.g. UCSC)	N/A

Methodology

Replicates	Experiments were repeated and results agree with each other.
Sequencing depth	Total reads: 124 million; uniquely-mapped reads: 79.6 million; length of reads: 76, single-end
Antibodies	Abcam #181111
Peak calling parameters	sh $SICERDIR/SICER.sh sample.bed control.bed mm10 1 200 142 0.74 600 .01
Data quality	4439 peaks at FDR 5%. Most are very broad peaks, so the mean enrichment fold is not very high.
Software	Sicer

Flow Cytometry

Plots

Confirm that:

☒ The axis labels state the marker and fluorochrome used (e.g. CD4-FITC).

☒ The axis scales are clearly visible. Include numbers along axes only for bottom left plot of group (a 'group' is an analysis of identical markers).

☐ All plots are contour plots with outliers or pseudocolor plots.

☐ A numerical value for number of cells or percentage (with statistics) is provided.

Methodology

Sample preparation	Primary MEFs from Myc-Egfp+/+ mice were treated with 20 µM of MG-132 for 6 h to inhibit proteasome degradation of MYC and therefore enhance the MYC-GFP signal, and then sorted for low- and high-GFP cells.
Instrument	BD FACSAria Violet
Software	The results were analyzed by Flowjo software.
Cell population abundance	Five percent of high-MYC and low-MYC MEFs were collected, and post-sorting analysis was done with the sorter. Immunostaining was used to validate the relative levels of MYC expression.
Gating strategy	The MG-132 untreated MYC-GFP cells were used as negative control for gating. Comparable mean FSC were set for both low-MYC and high-MYC population.

☒ Tick this box to confirm that a figure exemplifying the gating strategy is provided in the Supplementary Information.
